# Authors must follow the editorial guidelines on the use of large language models in review papers

**DOI:** 10.1007/s00210-025-04102-1

**Published:** 2025-03-29

**Authors:** Roland Seifert, Erik Hartman, KeWei Wang, Daniela Yildiz

**Affiliations:** 1 Institute of Pharmacology, Hannover Medical School, Carl-Neuberg-Str. 1, 30625 Hannover, Germany; 2 Department of Clinical Sciences, Lund University, Lund, Sweden; 3 Department of Pharmacology, Qingdao University, Qingdao, China; 4 Molecular Pharmacology, University of the Saarland, Saarbrücken, Germany

*Naunyn-Schmiedebergs Archives of Pharmacology* was one of the first journals to respond to paper mills by rigorously retracting suspicious papers (Seifert 2021). With the advent of artificial intelligence (AI), we intend to take a similarly rigorous approach to address the malicious use of large language models (LLMs) in scientific publishing.

Paper mills (also referred to as criminal science publishing gangs) sell fake papers with the purpose of making money in exchange for advancing the “academic career” of their customers by publishing papers in academic journals (Wittau and Seifert 2024; Wittau et al. 2024). This is a fundamental breach of the basis of science that builds on trust between authors, editors, referees, publishers, readers, and the public. Without mutual trust, science will be jeopardized with detrimental consequences (Sabel and Seifert 2021).

*Naunyn-Schmiedebergs Archives of Pharmacology* implemented a policy of mandating data deposition and requesting the following compulsory statement in published papers: The author confirm that all data were generated in-house and that no paper mill was used.

More recently, we implemented post-publication review of all images in papers published in *Naunyn-Schmiedebergs Archives of Pharmacology* (van Diest et al. 2025). This approach has already resulted in several paper retractions because of image manipulation and will continue to do so.

With the advent of effective LLMs and other AI tools, we and others (Liverpool 2023) anticipated an increase in LLM-generated papers produced by paper mills and other malevolent actors. However, it is very difficult to pinpoint and detect AI-generated content and thereby hinder fraudulent research from being published. Although tools exist which aim to detect whether text has been produced by LLMs, such tools produce false positives (Habibzadeh 2023) and have been shown to be biased towards non-native English writers (Liang et al. 2023). Of course, we want to avoid false accusations.

Starting mid 2023, we observed a large increase of submissions of review papers to *Naunyn-Schmiedebergs Archives of Pharmacology*. In some review papers, referees noted inadequate reference lists with key references missing, rather generic language and overt scientific errors. Such papers were rejected. But numerous submitted review paper underwent uneventful peer review, typically with two or three referee reports and one round of revision. In post-publication analysis, we suspect that some of these papers have largely been generated by AI. However, as mentioned, it is difficult to be certain that these papers are the result of honest scientific activity or the product of malicious practices fueled by AI.

For review papers, we welcome submissions from experts in their respective research fields and encourage contributions that highlight recent advancements, provide clear insights into future perspectives and research directions. Therefore, as a precaution and to facilitate possible later actions such as issuing notes of concern or retractions, effective in late 2023, The Editor-in-Chief, in consensus with the publisher Springer Nature, implemented the policy that all review papers published in *Naunyn-Schmiedebergs Archives of Pharmacology* must bear the following statement:

The authors confirm that no paper mill and no artificial intelligence was used.

All papers that are raised to us as an issue of concern are investigated by the Research Integrity Group of Springer Nature. The message of this Editorial to authors is very clear: we uphold the highest level of editorial scrutiny and sooner or later, you will be caught if you use AI technology in a review paper. We will rigorously and quickly investigate each suspicious paper that comes to our attention and will swiftly move on to retraction.

We kindly ask that readers report any suspicious papers directly to the Editor-in-Chief for investigation.

Finally, we should like to emphasize that compared to other journals, the retraction rate in *Naunyn-Schmiedebergs Archives of Pharmacology* is low (Wittau and Seifert 2024; van Diest et al. 2025), reflecting the high scientific standards that we aim to maintain.

## Data Availability

All source data for this work (or generated in this study) are available upon reasonable request.

## References

[CR1] Habibzadeh F (2023) GPTZero performance in identifying artificial intelligence-generated medical texts: a preliminary study. J Korean Med Sci 38(38):e319. 10.3346/jkms.2023.38.e319 37750374 10.3346/jkms.2023.38.e319 PMC10519776

[CR2] Liang W, Yuksekgonul M, Mao Y, Wu E, Zou J (2023) GPT detectors are biased against non-native English writers. Patterns 4(7):100779. 10.1016/j.patter.2023.100779 37521038 10.1016/j.patter.2023.100779 PMC10382961

[CR3] Liverpool L (2023) AI intensifies fight against ‘paper mills’ that churn out fake research. Nature 618(7964):222–223. 10.1038/d41586-023-01780-w 37258739 10.1038/d41586-023-01780-w

[CR4] Sabel BA, Seifert R (2021) How criminal science publishing gangs damage the genesis of knowledge and technology-a call to action to restore trust. Naunyn Schmiedebergs Arch Pharmacol 394(11):2147–2151. 10.1007/s00210-021-02158-3 34554267 10.1007/s00210-021-02158-3 PMC8514363

[CR5] Seifert R (2021) How Naunyn-Schmiedeberg’s Archives of Pharmacology deals with fraudulent papers from paper mills. Naunyn Schmiedebergs Arch Pharmacol 394(3):431–436. 10.1007/s00210-021-02056-8 33547901 10.1007/s00210-021-02056-8 PMC7865115

[CR6] van Diest RA, Seifert R, van der Heyden MAG (2025) An extra pair of eyes: adopting innovative approaches to detect integrity issues in Naunyn-Schmiedeberg’s Archives of Pharmacology. Naunyn Schmiedebergs Arch Pharmacol 398(1):1–8. 10.1007/s00210-024-03697-1 39751822 10.1007/s00210-024-03697-1 PMC11787271

[CR7] Wittau J, Seifert R (2024) How to fight fake papers: a review on important information sources and steps towards solution of the problem. Naunyn Schmiedebergs Arch Pharmacol 397(12):9281–9294. 10.1007/s00210-024-03272-8 38970685 10.1007/s00210-024-03272-8 PMC11582211

[CR8] Wittau J, Celik S, Kacprowski T, Deserno TM, Seifert R (2024) Fake paper identification in the pool of withdrawn and rejected manuscripts submitted to Naunyn-Schmiedeberg’s Archives of Pharmacology. Naunyn Schmiedebergs Arch Pharmacol 397(4):2171–2181. 10.1007/s00210-023-02741-w 37796310 10.1007/s00210-023-02741-w PMC10933159

